# Age-related changes in decision making with different wayfinding strategies

**DOI:** 10.1007/s00426-025-02195-0

**Published:** 2025-11-11

**Authors:** Ju-Yi Huang, Daniel Memmert, Oezguer A. Onur, Otmar Bock

**Affiliations:** 1 Institute of Exercise Training and Sport Informatics, German Sport University Cologne, Am Sportpark Müngersdorf 6, 50933 Cologne, Germany; 2 Department of Neurology, Faculty of Medicine and University Hospital Cologne, University of Cologne, Cologne, Germany

**Keywords:** *Cognitive aging*, *Spatial navigation*, *Wayfinding, decision-making*

## Abstract

Wayfinding skills are known to decay in older age. The present study investigated the differential effects of older age on five cognitive strategies that travelers can use for decision-making at intersections. To avoid interindividual and methodological biases, we used a within-person approach, and designed similar environments for all strategies. Thirty young and thirty older adults were asked to navigate five mazes that required decision-making by either the serial order strategy, the associative cue strategy, the beacon strategy, the relative location strategy, or the cognitive map strategy. The order of the five mazes was counterbalanced using a Latin square design; to reduce fatigue, the mazes were administered over two separate sessions. In agreement with extant research, we found that older participants’ wayfinding accuracy was poorer than that of young ones. Contrary to literature, however, this age-related decrement was not more pronounced for the cognitive map strategy than for the serial order and the associative cue strategy. We also found that the correlation between wayfinding performance with different strategies decreased to virtually zero in older age. Further regarding the cognitive map strategy, we found that older adults showed reduced ability to acquire incidental knowledge during wayfinding, but with no evidence that they compensated for these deficits by relying on auxiliary environmental cues. We interpret this pattern of findings as evidence that age-related wayfinding deficits are sensitive to task difficulty and are associated with a disintegration of the cognitive mechanisms involved in wayfinding, particularly in tasks with high visuospatial demands and multitasking requirements.

## Introduction

The ability to find our way in built environments is crucial for our independence and quality of life. Since wayfinding is a highly complex cognitive skill (Hegarty et al., 2022; Wolbers & Hegarty, 2010), age-related cognitive decline can hinder older persons to successfully navigate in unfamiliar buildings or cities. This study focuses on how aging affects one particular component of wayfinding: deciding in which direction to proceed across intersections encountered along the way.

Literature identified five cognitive strategies for such decision-making. The *serial order strategy* involves the recall of a memorized direction sequence, e.g., “first right, then straight on, then right again, …” (Iglói et al., 2009; Tlauka & Wilson, 1994). The *associative cue strategy* consists of the recall of memorized cue-direction associations, e.g., “turn left at the gas station” (Tlauka & Wilson, 1994; Waller & Lippa, 2007). The *beacon strategy* is only usable for destinations near a widely visible object; it involves moving closer to the visible object at each intersection (Waller & Lippa, 2007). The *relative location strategy* is only usable for destinations that can be triangulated from multiple widely visible objects; it consists of moving closer to the triangulated location at each intersection (Jacobs et al., 1997; Morris, 1984). Finally, the *cognitive map strategy* involves the formation of a mental representation of the environment and of one’s own position and orientation within it; this internal representation can be used to plan ones’ way through the environment, and to concurrently update ones’ own position and orientation (O’Keefe & Nadel, 1978; Tolman, 1948). Note that the latter two strategies require an allocentric reference frame, where directions and distances are represented relative to each other and independent of the traveler’s own position and orientation. In contrast, the former three strategies need only an egocentric frame, where directions and distances are represented relative to the traveler’s momentary position and orientation.

In everyday life, wayfinding can often be achieved by more than one of the above strategies. In such situations, participants can switch between strategies on repeated trips or even within a given trip (Hamburger, 2020; Wolbers & Hegarty, 2010), and they can use multiple strategies concurrently to enhance their wayfinding accuracy (Bock et al., 2023). To study the five strategies in isolation, we have recently developed a set of five virtual mazes, with each maze requiring the use of one of the aforementioned five strategies (Bock et al., 2024). Young participants took one guided and five self-guided trips through each maze, and we calculated the correlations between their accuracy on each pair of self-guided trips. We found that correlations between trips through the same maze were significantly higher than those between trips through different mazes, which supports the view that wayfinding decisions involved strategy-specific mechanisms. Furthermore, correlations between trips through different mazes were not significantly lower if one but not the other maze required an allocentric reference frame, which provides no support for a frame-specific decision mechanism. Finally, we found that correlations between trips through different mazes that required different reference frames were significantly higher than zero, which supports the existence of a generalized decision mechanism not limited to particular strategies or reference frames.

Wayfinding abilities are known to decline in older age (for reviews, see Lester et al., 2017; Moffat, 2009). It has been proposed that this age-related decline is more pronounced for strategies that require an allocentric reference frame (Hegarty et al., 2022; Moffat, 2009), and accordingly, older adults exhibited wayfinding problems most consistently for tasks requiring the cognitive map strategy (Gazova et al., 2013; Fricke & Bock, 2018; Head & Isom, 2010; Iaria et al., 2009; Liu et al., 2011), and less so for tasks requiring the serial order or the associative cue strategy (Fricke & Bock, 2018; Head & Isom, 2010; Liu et al., 2011; Wiener et al., 2012; Zhang et al., 2021; Zhong & Moffat, 2016). Age deficits were small or absent for tasks requiring the beacon or the relative location strategy (McAvan et al., 2021; Wiener et al., 2013).

Note that the preceding paragraph inferred a differential age-sensitivity of different wayfinding strategies based on between-study comparisons. Such comparisons may be biased by methodological and interindividual differences. Thus, methodological factors such as the number of intersections per trip (Bock et al., 2023), the number of alternatives per intersection (Bock & Huang, 2024), cue elevation (Durteste et al., 2023) and cue familiarity (Hamburger & Röser, 2014) are known to modify wayfinding difficulty, and individual factors such as sex, age, and habitual anxiety also influence wayfinding performance (Hegarty et al., 2022). As a consequence, actual age differences may be enhanced or attenuated by methodological details and by sample characteristics or interindividual factors.

To overcome this bias, the present study compared the wayfinding accuracy of young and older persons in all five strategy-specific mazes in a *within-person* approach, and using a *similar interior design* for all mazes. The purpose of the present work was fourfold.

H1: Replication hypothesis – We wanted to find out whether our within-person approach will replicate the differential age-sensitivity inferred from earlier studies. We hypothesized that, given the aforementioned role of interindividual and methodological differences, we may not necessarily yield the same age-sensitivity pattern.

H2: Dedifferentiation hypothesis – We were interested to know whether evidence for strategy-specific and for generalized wayfinding decision mechanisms, yielded before in young adults (Bock et al., 2024), will emerge in older adults as well. To find out, we conducted the same correlation analyses with new data and compared between ages. It has been shown that with advancing age, distinct cognitive functions may rely increasingly on common rather than on function-specific processes – a phenomenon known as cognitive dedifferentiation. For example, decision-making in older adults tends to rely more on generalized than on decision-specific mechanisms (Hülür et al., 2015; la Fleur et al., 2018). We therefore hypothesized that this cognitive dedifferentiation will increase the evidence for generalized mechanisms, at the expense of strategy-specific mechanisms.

H3: Resource competition hypothesis – Earlier dual-task research documented that wayfinding can compete with another task for a common pool of resources already in young adults (Fang et al., 2020; Meilinger et al., 2008); this competition might be even more pronounced in older age since dual-task costs often increase with aging (Beurskens & Bock, 2012; Shumway-Cook & Woolacott, 2000). We therefore reasoned that, if acquisition of incidental spatial knowledge constitutes a separate task that is distinct from the wayfinding task, then the two tasks may interfere and the interference may be more pronounced in older age. In other words, we hypothesized that the acquisition of incidental spatial knowledge while wayfinding may be degraded in older adults compared to young ones.

H4: Compensation hypothesis – We wanted to know whether older participants partly compensate for their wayfinding deficits by exploiting auxiliary spatial cues that cannot be completely avoided even in highly controlled experimental scenarios. The use of auxiliary cues like signage and maps has been called ‘aided wayfinding’ (Wiener et al., 2009), and earlier research suggests that it ameliorates age-related wayfinding deficits (Xu et al., 2024). We reasoned that our strategy-specific mazes also provide auxiliary spatial cues: persons who notice that they are in the southwest corner of a maze can infer that their destination cannot be further south or further west. To determine whether participants indeed utilized such auxiliary cues, we administered a test that assessed their knowledge of spatial locations *without* the auxiliary cues present during wayfinding. Since older persons are known to compensate for their cognitive deficits in multiple ways (e.g., Tomaszewski Farias et al., 2018), we hypothesized that they also will compensate for impaired spatial knowledge by utilizing auxiliary cues, so that the difference between wayfinding performance and spatial knowledge scores will be larger in older compared to young participants.

## Method

### Participants

Thirty older adults from 60 to 80 years of age were recruited through notices at local universities and communities, word of mouth, and written postings. All were healthy by self-report, and those who wore eyeglasses in their daily life continued to wear them during testing. None of the participants dropped out. The demographic characteristics are summarized in Table 1. The data of 30 young adults came from an earlier study (Huang et al., 2025); they were not registered anew to avoid an unnecessary duplication. The purpose of that earlier study was to compare performance in three groups of young participants, not to compare performance in young and older persons as in the present work. One group in that earlier study was tested using the same software, protocol and instructions as in the present work, and their data were therefore now used for our main analysis. A second group differed only in that the decision time at intersections was constrained to 3 s rather than being unlimited, and their data were now used for our additional analysis. A third group further differed in that no error corrections were required, and their data were not considered in the present work. Written informed consent was obtained from all participants before testing began. This study was part of a research program that was approved by the Ethics Commission of the German Sport University, and all procedures were in compliance with the Declaration of Helsinki.

**Table 1:** Demographic characteristics of participants

	young adults	older adults
sample size	30	30
age (mean years ± SD)	24.5 ± 2.5	69.8 ± 7.2
female/male	15/15	16/14
education (mean years ± SD)	15.3 ± 1.5	12.8 ± 4.0
ASKU	10.07 ± 2.3	10.93 ± 2.4

Note. *ASKU* Short Scale for Measuring General Self-Efficacy Beliefs (in German: Allgemeine Selbstwirksamkeit Kurzskala)

### Procedures

All participants initially completed a demographics questionnaire as well as the self-efficacy scale (ASKU, Beierlein et al., 2013). To assess self-efficacy for navigation ability rather than overall self-efficacy, we modified the three original ASKU items to (1) Can you rely on your sense of orientation even in difficult situations? (2) Do you usually find your way in unfamiliar places without asking for help? (3) Are you typically able to navigate even in complex situations? As with the original ASKU, participants responded on a 5-point scale ranging from 1 = “not at all” to 5 = “perfectly”, and the total score was obtained by adding up the scores of all items such that it could range from 3 to 15.

Following the ASKU questionnaire, participants performed the wayfinding task, which involved six consecutive trips through each of five strategy-specific mazes. After the last trip through each maze, participants performed a direction judgment task. In this task, participants were given a computer-drawn illustration showing a central cross, a sketched human head (oval plus nose) centered about the cross, and a peripheral circle of 14.5 cm in diameter also centered about the cross. They were instructed: ‘*This head represents your position and orientation right now*,* at the end of your last trip. Please draw a line from the center of the head (i.e.*,* the cross) to the outer circle*,* pointing toward the starting point of your last trip.*’ Direction judgment accuracy was quantified as the absolute angle between actual and drawn directions, ranging from 0° to 180°. This task quantifies incidental spatial knowledge since knowing the direction from the end to the start of the last trip was not needed for successful completion of the wayfinding task.

In addition, the last trip in maze C was followed by the maze recreation task. In this task, participants were given a schematic map of maze C, along with individual images of the visual cues they had encountered in that maze; those images were provided in a mixed order, not in the sequential order in which the cues were encountered in maze C. They were instructed: ‘*Please place each cue at the correct intersection on the map. Once a cue was placed*,* it could not be removed; however*,* you were allowed to place another cue on top of it if needed*.’ Maze recreation accuracy was quantified as the proportion of correctly placed cues, ranging from 0 = “no cue placed correctly” to 1 = “all cues placed correctly”.

To avoid fatigue, each participant was given the above-mentioned tasks in two sessions, each lasting 40 to 60 min. The interval between sessions ranged from two to seven days, depending on participants’ availability. The mazes were presented using a Latin square design to systematically balance their order across participants, with two mazes presented in the first session and the remaining three in the second session. All participants completed the questionnaire, the five mazes, and additional tasks.

### Wayfinding task in general

**Fig. 1:**
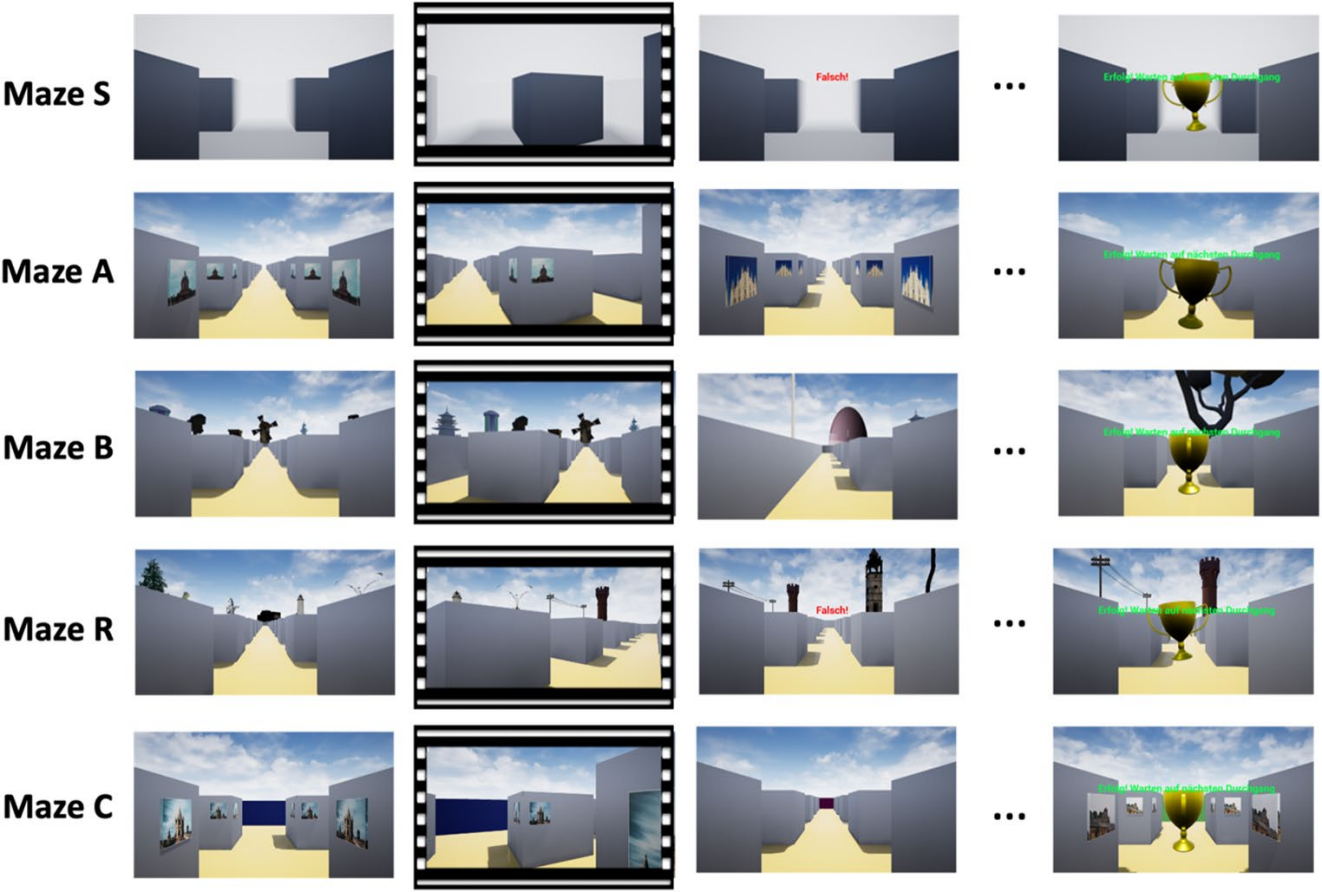
Example snapshots of a chain of event from a trip through each maze. Note: The first column illustrates the stops at intersections for decision given in each maze. The second column, marked with sprocket holes, indicates passive movement from one intersection to the next after the correct direction is given. The third column shows stops at the next intersection, and for Maze S and R, it illustrates an error message when an incorrect direction is given, after which participants had to try again at the same intersection. Maze S requires a serial order strategy, Maze A an associative cue strategy, Maze B a beacon strategy, Maze R a relative location strategy, and Maze C a cognitive map strategy

We used the virtual reality platform Unreal Engine^®^ (Epic Games, Inc., Cary, NC) to render virtual mazes on a 16” laptop PC screen, and to record participants’ responses. In each maze, participants were passively transported to an intersection of two corridors, where they stopped. They then indicated their decision to either proceed straight on, to turn left, or to turn right by deflecting a joystick handle in the corresponding direction. If their response was incorrect, a red error message (“Falsch!”) displayed on screen for 5 s, and participants then could try again (see details in next section for what constitutes “correct” in the different mazes). If their response was correct, they were passively transported along the chosen corridor to the next intersection. This chain of events was repeated until participants reached the last intersection, where a trophy was displayed to indicate completion of a trip. Participants were then returned to the starting location for the next trip. Figure 1 shows an example of a chain of events from a trip through each maze.

Each participant undertook six trips through each given maze. On the first trip, the experimenter told them at each intersection in which direction to proceed. On the subsequent five trips, participants had to decide on their own. They were asked to respond at their own pace without hurrying.

Performance on each self-guided trip was quantified as response accuracy, representing the proportion of intersections on which participants responded correctly on their first attempt. The wayfinding accuracy could range from 0 = “no intersection correct on first attempt” to 1 = “all intersections correct on first attempt”.

### Wayfinding task for strategy-specific mazes

Each of the five mazes was designed to enforce one particular wayfinding strategy. Before starting on a given maze, participants were instructed which strategy they should use to succeed.

Maze S (Serial order strategy): Participants had to follow the same route through twelve intersections on every trip. All intersections appeared visually identical, with no distinctive cues (see Fig. 1, maze S). Successful navigation therefore required memorizing the exact sequence of directional choices – for example, “turn right at the first intersection, left at the second,” and so on.

Maze A (Associative cue strategy): Each of the twelve intersections featured a unique visual cue displayed at all corners (e.g., Milan Cathedral in Fig. 1, maze A). Each cue was consistently associated with a specific turn direction, but was presented in a different order on each trip. For instance, Milan Cathedral might appear at the third intersection on one trip and at the ninth intersection on another trip, but in both cases, it signaled a right turn. Participants therefore had to memorize cue-direction associations (e.g., “Milan Cathedral → right turn”), not a fixed sequence of turns (e.g., not “third intersection → right turn”).

Maze B (Beacon strategy): Participants were told to navigate directly toward an exotic-looking tree that marked the goal. This tree was one of thirteen tall landmarks placed equidistantly around the maze and visible from all locations (e.g., Fig. 1, last column in maze B). Since intersections lacked local cues and the tree’s position with respect to the maze varied across trips, successful performance required reducing the distance to the visible tree at each intersection.

Maze R (Relative location strategy): This maze resembled maze B in structure, but the goal was defined as an imaginary location forming an equilateral triangle with two of the thirteen visible tall objects (i.e., tower and poles in maze R). As in maze B, the landmarks changed position across trips. To succeed, participants had to mentally triangulate the goal’s location and reduce their distance to it at each intersection.

Maze C (Cognitive map strategy): Participants were instructed to visit twelve visual cues. The maze formed a 5 × 7 grid in which intersections with cues on the walls alternated with intersections without cues (see Fig. 1, maze C). At each cued intersection, the next-to-visit cue was provided on a sheet of paper; it was always two intersections away, requiring only one directional decision. The cues had to be visited in a different order on each trip and therefore could not be associated with fixed turn directions, hence neither the serial order nor the associative cue strategy was applicable. Instead, participants had to mentally represent the spatial layout of the cues, monitor their own position and orientation, and make decisions accordingly — thus relying on a cognitive map. To support self-orientation, each external wall of the maze was painted a different color.

### Data analysis

We assessed the normality of the distribution of wayfinding accuracy scores using the Shapiro-Wilk test. Results indicated significant deviations from normality in 14 out of 25 trips for the older adults and in all 25 trips for the younger adults (all *p* < 0.05). We therefore analyzed wayfinding accuracy with non-parametric statistics, using the R package *nparLD*, tailored for the *F2-LD-F1* model (Noguchi et al., 2012). Wald-type tests were chosen for their robustness in handling non-parametric data, and their suitability for analyzing multiple independent variables in repeated-measures designs.

To address the replication hypothesis (H1), a Wald-type test was conducted for each maze with the dependent variable ‘accuracy’, the within-factor ‘trip’ (2, 3, 4, 5, 6), and the between-factors ‘age’ (young, older) and ‘sex’ (male, female), to determine whether wayfinding performance in a given maze depends on age at least on some trips. Additionally, a Wald-type test was conducted with the dependent variable ‘average accuracy’ (across all five self-guided trips), the within-factor ‘maze’ (S, A, B, R, C), and the between-factors ‘age’ and ‘sex’, to find out whether age differences varied across mazes.

The dedifferentiation hypothesis (H2) was addressed in the same correlation analysis as in Bock et al. (2024). Mutually independent^1^ Spearman correlations between trips (see Table 8. to 10 in the Appendix), were subdivided into three sets. Set r_within-maze_ represented correlations between trips through the same maze, set r_within-frame_ comprised correlations between trips through two different mazes, both requiring or both not requiring an allocentric reference frame, and r_between-frames_ represented correlations between trips through two mazes, one requiring and the other not requiring an allocentric reference frame. Since three correlations from young adults could not be calculated due to ceiling effects in maze R (see Table 8.), we conducted an additional analysis as a precautionary measure, using the data from the same earlier study (Huang et al., 2025) but from another group of young adults. Procedures in that group differed only in that the decision time at intersections was limited: participants had to respond within 3 s to avoid an error message. This time constraint reduced the incidence of ceiling effects, such that all correlations could be calculated (see Table 10).

Each set of correlations was transformed using Fisher’s Z transformation and compared between ages by Mann–Whitney U test from the R package *wilcox.test*. Thus, r_within-maze_ of older adults was compared to r_within-maze_ of young adults, and accordingly for r_within-frame_ as well as for r_between-frames_. Since maze B and R probably required little decision-making (Bock et al., 2024), the comparisons were repeated after eliminating data from those two mazes.

To address the resource competition hypothesis (H3), errors in the direction judgment task were analyzed by a Wald-type test with the dependent variable ‘absolute error’, the within-factor ‘maze’ (S, A, B, R, C) and the between-factors ‘age’ and ‘sex’. Post-hoc Dunn’s tests were performed to decompose a significant ‘maze x age’ term by comparing ages within each maze.

The compensation hypothesis (H4) was addressed by a Wald-type test with the dependent variable ‘accuracy’, the within-factor ‘task’ (last trip in maze C, maze recreation task) and the between-factors ‘age’ and ‘sex’. According to the hypothesis, the ‘age x task’ term should be significant. This analysis controlled for the potentially different variances in the two tasks by normalizing the scores from either task using

accuracy_norm_ = (accuracy – accuracy_mean_)/SD + accuracy_mean_ (1),

where accuracy_mean_ is the across-participant mean for a given task and age group, and SD is the pooled standard deviation for a given task, calculated from the standard deviations of each age group.

Since standard effect sizes like partial eta² are not applicable to rank-based methods such as the Wald-type statistic, we report partial eta² (η²p) values derived from linear mixed-effects models with the same structure as used in the Wald-type tests. These values should be interpreted with caution, however, as the underlying data are not normally distributed. For the pairwise comparisons, we calculated effect size as Cliff’s Delta (δ), which is suitable for non-normally distributed data (Macbeth et al., 2011).

## Results

### H1: Replication hypothesis – age differences in wayfinding performance

**Fig. 2:**
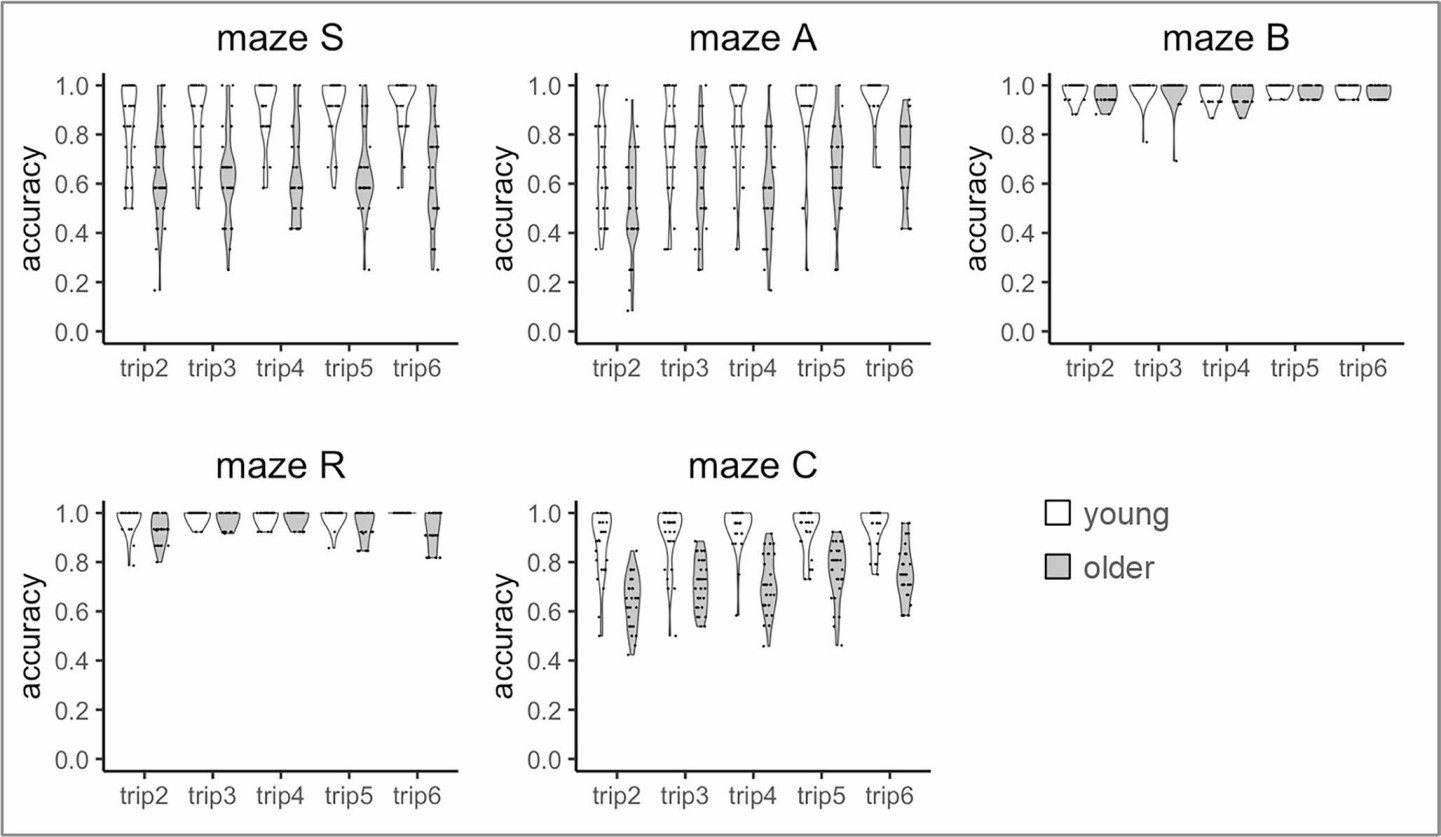
Distribution of accuracy across each self-guided trip for each maze and age group. Note: Each dot represents one participant; the width of the violin around the dots indicates the data distribution density. Trip 1 is not shown as it was guided by the experimenter.

Figure 2 illustrates participants’ wayfinding accuracy on self-guided trips through each of the five mazes, and Table 2 summarizes the Wald-type tests for each maze. Accuracy increased from trip to trip in all mazes, and young participants outperformed older ones in all mazes. Sex differences did not reach statistical significance in maze A, B and C, but showed a marginal significance in maze S and R, where males performed better than females across trips. It is conceivable that in a larger sample, this marginal significance would either disappear or extend to the other mazes as well. Table 2 further reports no significant interaction, except for ‘age x trip’ in maze A and R, where the trip-to-trip increase in accuracy was more pronounced in young compared to older persons.

**Table 2:** Wald-type test outcome for each maze

	statistic	df	*p*-value	η²_*p*_
maze S				
age	39.40	1	**< .001**	0.070
sex	3.37	1	.067	0.040
trip	18.24	4	**.001**	0.100
age x sex	0.44	1	.508	0.009
age x trip	6.70	4	.153	0.020
sex x trip	8.06	4	.089	0.010
age x sex x trip	2.00	4	.736	0.007
**maze A**				
age	33.50	1	**< .001**	0.020
sex	0.82	1	.367	0.001
trip	80.14	4	**< .001**	0.310
age x sex	0.56	1	.455	<0.001
age x trip	13.23	4	**.010**	0.020
sex x trip	2.06	4	.724	<0.001
age x sex x trip	8.32	4	.081	0.001
**maze B**				
age	8.34	1	**.004**	0.008
sex	0.13	1	.717	<0.001
trip	22.15	4	**< .001**	0.003
age x sex	1.54	1	.215	<0.001
age x trip	2.78	4	.595	0.002
sex x trip	3.53	4	.473	<0.001
age x sex x trip	2.54	4	.638	0.001
**maze R**				
age	29.06	1	**< .001**	<0.001
sex	4.17	1	**.041**	0.007
trip	18.82	4	**.001**	<0.001
age x sex	0.00	1	.986	0.008
age x trip	20.53	4	**< .001**	0.010
sex x trip	7.68	4	.104	0.002
age x sex x trip	4.25	4	.374	0.010
**maze C**				
age	92.27	1	**< .001**	0.290
sex	1.32	1	.251	0.020
trip	50.50	4	**< .001**	0.230
age x sex	1.33	1	.248	0.020
age x trip	4.64	4	.326	0.020
sex x trip	5.41	4	.247	0.010
age x sex x trip	3.43	4	.488	0.006

Note. *η*²*p* (partial eta squared) represents the effect size, showing how much of the variance in the results can be explained by a specific factor, after accounting for other variables

**Fig. 3:**
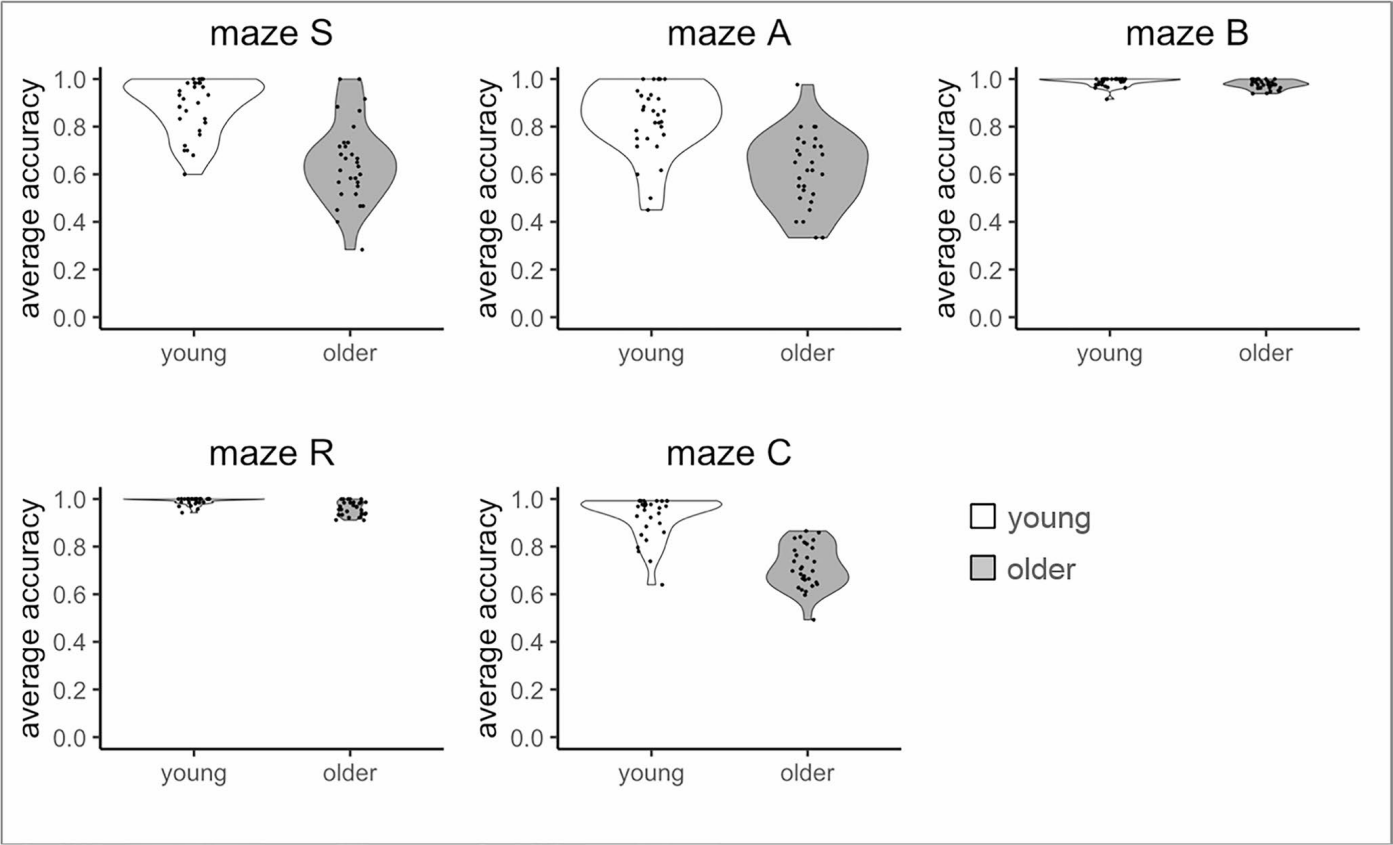
Distribution of average accuracy across trips for each maze and age group. Note: Each dot represents one participant; the width of the violin around the dots indicates the data distribution density.

The Wald-type test with ‘maze’ as a factor (see Table 3.) confirmed the higher accuracy of young compared to older participants, and additionally yielded significance for ‘maze’ and for ‘age x maze’. The latter significance implies that age differences were not the same for all mazes, and indeed, Fig. 3 illustrates that age differences were smaller in maze B (young: M = 0.99, SD = 0.02; older: M = 0.98, SD = 0.02) and R (young: M = 0.99, SD = 0.02; older: M = 0.96, SD = 0.03) than in the maze S (young: M = 0.89, SD = 0.12; older: M = 0.65, SD = 0.17), A (young: M = 0.83, SD = 0.15; older: M = 0.61, SD = 0.15) and C (young: M = 0.92, SD = 0.09; older: M = 0.72, SD = 0.09), due to a ceiling effect in both ages.

**Table 3.** Wald-type test outcome across all mazes

	statistic	df	*p*-value	η²_*p*_
age	94.19	1	**< .001**	0.650
sex	7.47	1	**.006**	0.060
maze	392.34	4	**< .001**	0.610
age x sex	0.54	1	.463	0.010
age x maze	27.63	4	**< .001**	0.250
sex x maze	3.19	4	.526	0.020
age x sex x maze	6.78	4	.148	0.008

To determine whether age differences in maze S and A were smaller than those in maze C, as stipulated by the replication hypothesis (see Introduction), we repeated the Wald-type test after excluding maze B and R. The ‘age x maze’ term was no longer significant (see Table 4), thus providing no support for the replication hypothesis.

**Table 4:** Wald-type test as in Table 3., but excluding maze B and R

	statistic	df	*p*-value	η²_*p*_
age	97.31	1	**< .001**	0.630
sex	4.20	1	**.04**	0.050
maze	19.60	2	**< .001**	0.150
age x sex	1.19	1	.274	0.020
age x maze	0.14	2	.933	0.003
sex x maze	0.97	2	.615	0.010
age x sex x maze	0.01	2	.997	0.001

Tables 3. and 4 further indicate a significant effect of sex, with males performing slightly better than females across mazes (male: M = 0.87, SD = 0.17; female: M = 0.84, SD = 0.17) and after excluding maze B and R (male: M = 0.75, SD = 0.17; female: M = 0.79, SD = 0.18).

### H2: Dedifferentiation hypothesis – age differences in wayfinding correlation

**Fig. 4:**
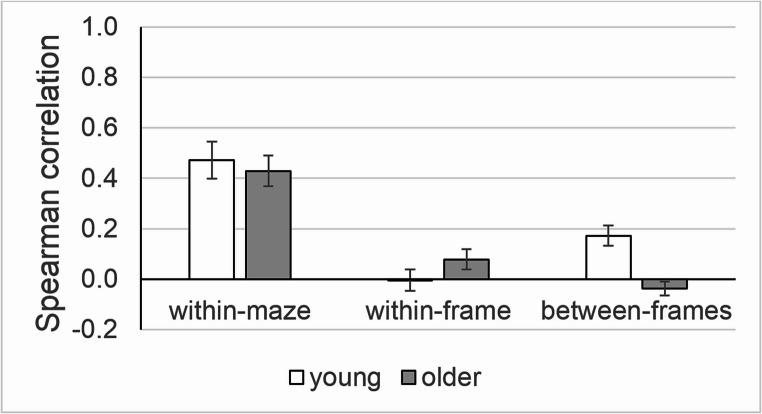
Correlations between the accuracy on different pairs of trips. Note: Bars are age group means, and whiskers are standard errors. Within-maze: correlations between trips through the same maze; within-frame: correlations between trips through different mazes with the same reference frame; between-frames: correlations between trips through different mazes with different reference frames.

Figure 4 illustrates the correlations r_within-maze_, r_within-frame_ and r_between-frames_, separately for young and older persons. The age difference did not reach statistical significance for r_within-maze_ (W = 219; *p* = .423; δ = 0.15) and for r_within-frame_ (W = 154; *p* = .341; δ = 0.20), but it was highly significant for *r*_*between-frames*_ (W = 101; *p* < .001; δ = − 0.60). These results indicate that older adults’ performance was consistent across trips within the same maze and across mazes within the same reference frame, similar to young adults. However, unlike young adults, older ones showed no appreciable consistency across mazes with different reference frames as r_between-frames_ was close to zero in this age group. Findings were quite similar in the supplementary analysis (data with a 3 s decision time for young adults). Again, the age difference was not significant for r_within-maze_ (W = 249; *p* = .192; δ = 0.25) and r_within-frame_ (W = 96; *p* = .239; δ = − 0.25), but was highly significant for r_between-frames_ (W = 76; *p* < .001; δ = − 0.74).

**Fig. 5:**
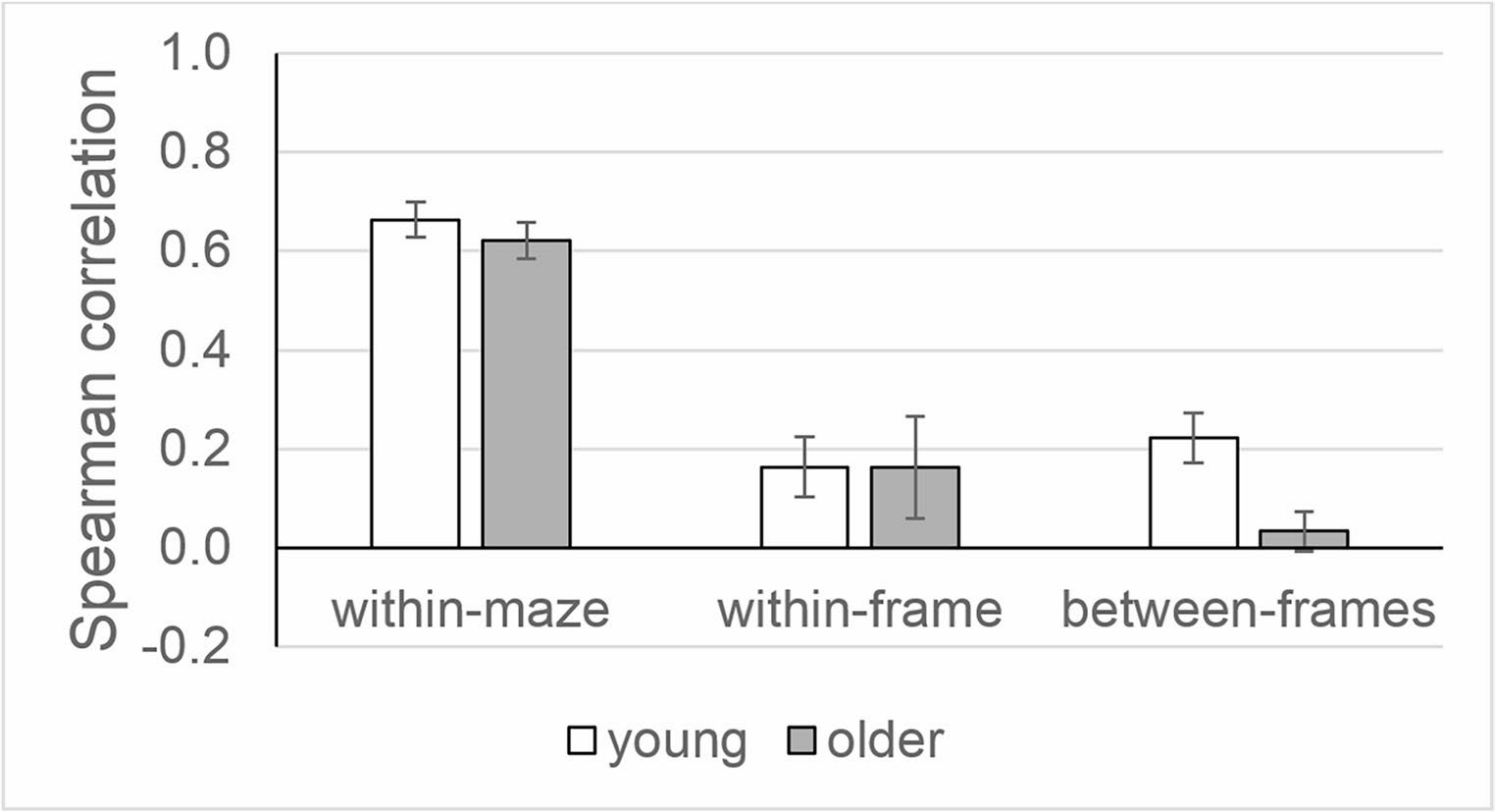
Correlations as in Fig. 4, but excluding maze B and R. Note: Bars are age group means, and whiskers are standard errors.

Figure 5 shows the corresponding data after excluding maze B and R. Again, the age difference was not significant for r_within-maze_ (W = 91; *p* = .291; δ = 0.26) and r_within-frame_ (W = 10; *p* = .686; δ = 0.25), but was significant for r_between-frames_ (W = 67; *p* = .023; δ = − 0.48). No supplementary analysis was necessary since all correlations could be calculated after excluding maze B and R. Summing up, these outcomes do not support the dedifferentiation hypothesis.

### H3: Resource competition hypothesis – age differences in the direction judgment task

**Fig. 6:**
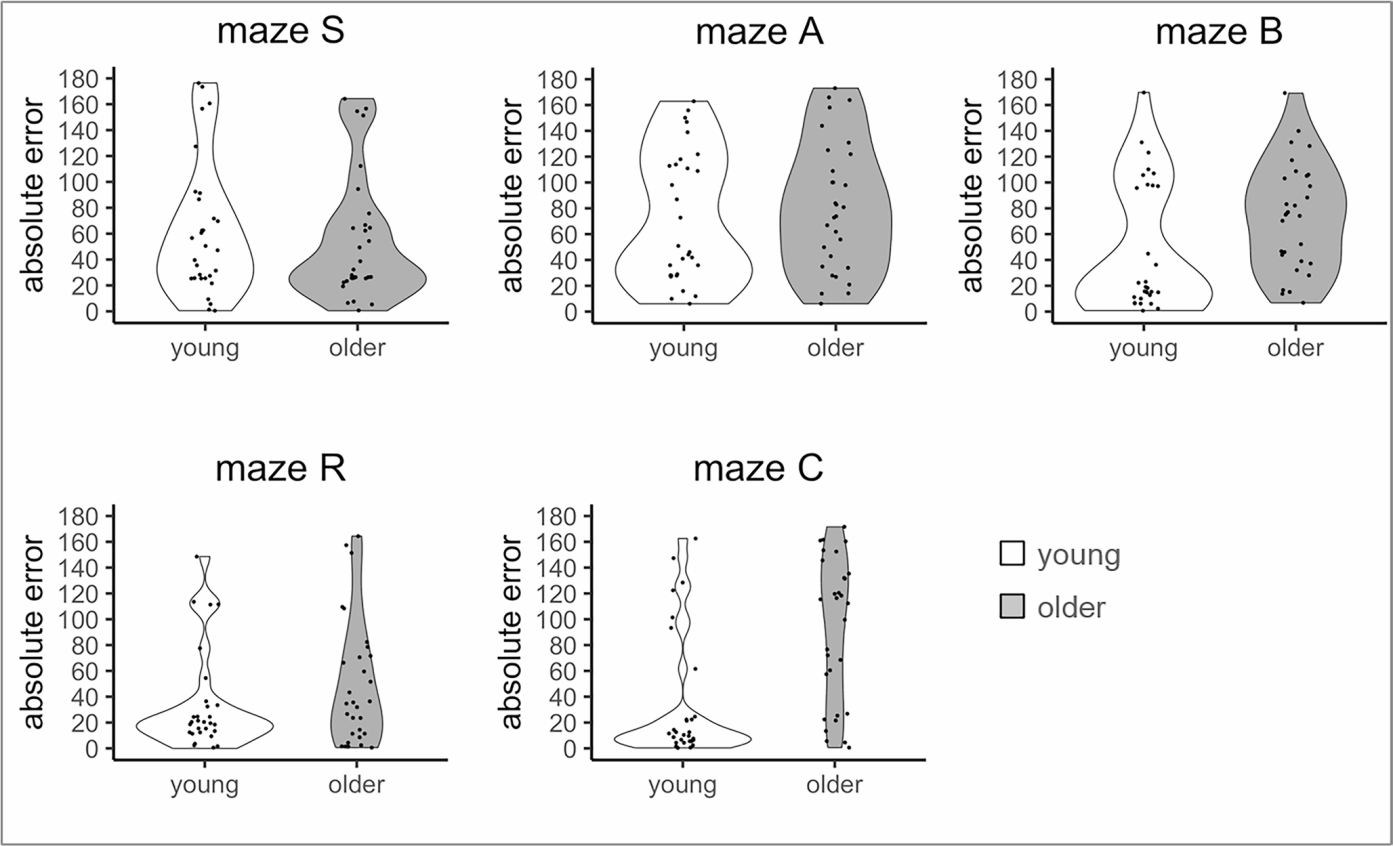
Distribution of error on the direction judgment task for each maze and age group. Note. Each dot represents one participant; the width of the violin around the dots indicates the data distribution density

Figure 6 depicts the distribution of absolute angular errors for each maze. The Wald-type test in Table 5. confirms that the age difference was significant, and that performance differed between mazes. In addition, the ‘age x maze’ and ‘sex x maze’ terms were significant, indicating that age- and sex-related differences in performance varied across mazes. Post-hoc Dunn’s tests with Bonferroni adjustments showed that the age difference was highly significant only in maze C (see Table 6), providing partial support for the resource competition hypothesis. Sex differences were not significant for any maze in the post-hoc tests (all *p* > .05)

**Table 5.** Wald-type test for the direction judgment task across all mazes

	statistic	df	*p*-value	η²_*p*_
age	11.77	1	**.001**	0.130
sex	0.06	1	.804	<0.001
maze	26.02	4	**< .001**	0.080
age x sex	0.07	1	.785	<0.001
age x maze	19.02	4	**.001**	0.070
sex x maze	10.67	4	**.031**	0.040
age x sex x maze	6.74	4	.150	0.030

**Table 6:** Dunn’s test outcome for the age differences in each maze

maze	statistic	*p*-value	*p*.adjusted	δ
maze S	0.67	.505	1.00	0.10
maze A	−0.66	.511	1.00	−0.10
maze B	−2.45	.014	.072	−0.37
maze R	−1.24	.214	1.00	−0.19
maze C	−3.78	< .001	**< .001**	−0.57

*Note. p*.adjusted is the Bonferroni corrected *p*-value. δ represents the effect size calculated using Cliff’s Delta

### H4: Compensation hypothesis – age differences in wayfinding and maze recreation task

The Wald-type test in Table 7 confirms that the age difference was significant and that performance differed between tasks. However, the ‘age x task’ term was not significant. This indicates that older adults exhibited lower accuracy than young adults, and the maze recreation task exhibited lower accuracy than the wayfinding task for maze C. However, task differences did not vary between young and older participants, thus providing no support for the resource compensation hypothesis.

**Table 7:** Wald-type test for wayfinding and maze recreation task for maze C

	statistic	df	*p*-value	η²_*p*_
age	4.70	1.00	**.030**	0.040
sex	1.68	1.00	.195	0.020
task	14.77	1.00	**<.001**	0.110
age x sex	0.33	1.00	.566	0.010
age x task	1.41	1.00	.236	0.050
sex x task	1.28	1.00	.258	0.002
age x sex x task	1.52	1.00	.218	0.030

## Discussion

The present study investigated the differential influence of older age on wayfinding in five strategy-specific virtual mazes. To avoid interindividual and methodological biases, we adopted a within-person approach, and used a similar interior design for all strategies. We hypothesized that we may not necessarily replicate earlier findings about age decrements being most pronounced for the cognitive map strategy (maze C), less so for the serial order and the associative cue strategy (maze S and A), and small or absent for the beacon and the relative location strategy (maze B and R), because findings can be biased by individual and methodological differences (replication hypothesis). We further hypothesized that the evidence for a generalized, strategy-overarching mechanism will be stronger in older persons compared to young ones (dedifferentiation hypothesis), that older adults will have fewer resources available for incidental learning in the wayfinding task (resource competition hypothesis), and that age-related wayfinding deficits are partly compensated by exploiting auxiliary spatial cues (compensation hypothesis).

Regarding the replication hypothesis (H1), we observed the smallest age-related decrements of wayfinding accuracy in maze B and R, which is in accordance with earlier studies (McAvan et al., 2021; Wiener et al., 2013), but we did not find larger decrements in maze C compared to maze S and A, which disagrees with earlier studies (Fricke & Bock, 2018; Gazova et al., 2013; Head & Isom, 2010; Iaria et al., 2009; Liu et al., 2011; Wiener et al., 2012; Zhang et al., 2021; Zhong & Moffat, 2016). We thus yielded partial support for the replication hypothesis, and conclude that larger age decrements in maze C may or may not emerge in dependence on methodological factors affecting task difficulty, and on individual factors such as sex and anxiety.

We further found wayfinding accuracy to be slightly higher in males than in females, mainly so for the relative location strategy, and marginally so for the serial order strategy. This finding aligns with the study of Dahmani et al. (2023), where males tended to outperform females in a navigation task that requires the computation or estimation of angles and distances, but not in a task that required navigation on defined routes. It is also in agreement with a number of studies that reported a male advantage on some but not on other spatial abilities, as summarized in a review article by New and Truxaw (2021).

Regarding the dedifferentiation hypothesis (H2), we found no age differences for r_within-maze_ or r_within-frame_, but we yielded significant age differences for r_between-frames_ which was about zero in older persons. Since r_within-maze_ is an indicator of strategy-specific mechanisms while r_between-frames_ is an indicator of a generalized mechanism (Bock et al., 2024), our data suggest that strategy-specific mechanisms were age resistant, while the generalized mechanism became ineffective in older age. This outcome is not consistent with the dedifferentiation hypothesis, and rather supports the disintegration hypothesis (Argiris et al., 2024; Fjell et al., 2017); this competing hypothesis states that cognitive aging is associated with a reduced correlation between performance scores on different tasks.

Regarding the resource competition hypothesis (H3), we reasoned that accuracy in the direction judgment task is an indicator of incidental learning, a phenomenon previously reported for a number of cognitive tasks, including wayfinding (Bock et al., 2024; van Asselen et al., 2006). We consider it to be incidental since knowing the direction to the starting point was not needed for successful wayfinding in any of our five mazes. Therefore, the observed age decrement in the direction judgments following maze C, but not following the other mazes, constitutes evidence in favor of the resource competition hypothesis. In particular, our data are in accordance with the view that our older participants were particularly challenged by maze C, which imposed higher cognitive demands. While maze S and A primarily required simple sequence or pair memorization, and maze B and R involved cue searching and location identification, maze C demanded both continuous monitoring of heading direction and the recall of multiple spatial cues and their interrelationships. This increased cognitive load likely depleted the limited cognitive resources in older adults, reducing the capacity available for incidental learning. It is indeed well established in multitasking literature that increasing the resource demand of one task degrades performance on a concurrent task (Maylor & Lavie, 1998; Salthouse et al., 1989).

Regarding the compensation hypothesis (H4), we found no evidence for a lower age decrement in the wayfinding task compared to the maze recreation task. This outcome does not support the view that older persons exploited auxiliary spatial cues more extensively than young ones, to compensate for their wayfinding deficits in maze C. However, it remains conceivable that older persons do compensate for their wayfinding deficits in real life where auxiliary cues are more abundant than in the present virtual-reality scenario (cf. Figure 1, maze C). It indeed has been shown that older persons compensate for various cognitive deficits in everyday life (e.g., Tomaszewski Farias et al., 2018).

A potential limitation of our study, like that of many earlier wayfinding studies, is that participants did not physically walk through the mazes; they rather were seated while a walk through the mazes was displayed on a computer screen. Therefore, the natural interplay between visual signals, vestibular-proprioceptive feedback and locomotor outflow was absent. This possibly degraded wayfinding accuracy because of reduced ecological validity, or enhanced it because concurrent processing of multiple sensory and motor signals was not necessary.

A second potential limitation is that, like in the majority of experimental research, our wayfinding scenarios lacked the familiarity, sense of purpose, distracting events and time pressures oftentimes present in everyday life. This reduction of ecological validity might also have degraded or enhanced wayfinding performance. A recent meta-analysis indeed reported smaller age-related decrements in real-world compared to virtual wayfinding (Xu et al., 2024), likely due to the abundance of auxiliary cues (Wiener et al., 2009); these real-world benefits were sometimes only modest (Taillade et al., 2016), suggesting that they may depend on the particular type, number, and distribution of auxiliary cues.

Finally, another potential limitation is that our findings may not necessarily generalize to wayfinding tasks whose difficulty differs substantially from the present ones. For example, age decrements may be different from those reported here when the number of intersections, the number of directional options at each intersection, or the ratio of intersections with and without visual cues is different from the present study. Future research should elucidate the role of these and other constraints on wayfinding, and on age-related decrements in wayfinding.

In conclusion, older age was associated with reduced wayfinding accuracy when using the serial order, associative cue, and cognitive map strategies (maze S, A, and C, respectively). This decline was paralleled by impairments in generalized, but not strategy-specific, wayfinding mechanisms, and by increased strain on visuospatial processing resources when wayfinding with the cognitive map strategy (maze C) – i.e., the strategy which likely imposes the highest visuospatial demands. Notably, here was no evidence that older adults compensated for their deficits by exploiting auxiliary environmental cues more efficiently than young adults did. Taken together, this pattern of findings suggests that age-related wayfinding impairments may be driven primarily by declines in domain-overarching cognitive functions, such as attention and executive control, rather than by strategy-specific deficits. This argues for an expanded focus in wayfinding training for older adults – one that includes not only the practice of specific strategies, but also the practice of overarching cognitive skills. If such training is implemented in virtual environments, photorealistic displays may not be necessary if auxiliary cues do not play a prominent compensatory role.

## Data Availability

The code for running the experiments and datasets generated and analyzed during the current study are available from the corresponding author on reasonable request.

## Appendix 1

For each pair column (e.g., trips-within_maze), the left column indicates the trips whose accuracy was correlated; for example, S2 – A3 refers to the correlation between the second trial (first self-guided trial) in maze S and the third trial in maze A. The respective right column provides the pertinent Spearman correlations. ‘NA’ indicates that a correlation could not be calculated because of ceiling effects.

Formatting as in Table 8, except that the ‘trips’ columns are not shown since they are identical to Table .

**Table 8.** Independent bivariate correlations between the accuracy on different trips in young adults

trips	*r*_within−maze_	trips	*r*_within−frame_	trips	*r*_between−frames_
S2 – S3	0.72	S2 – A3	0.12	S2 – R3	0.19
S3 – S4	0.77	S3 – A4	0.12	S3 – R4	0.35
S4 – S5	0.60	S4 – A5	0.34	S4 – R5	0.02
S5 – S6	0.64	S5 – A6	0.07	S5 – R6	NA
A2 – A3	0.78	S2 – B3	-0.23	S2 – C3	0.45
A3 – A4	0.79	S3 – B4	0.12	S3 – C4	0.16
A4 – A5	0.53	S4 – B5	-0.31	S4 – C5	0.15
A5 – A6	0.50	S5 – B6	-0.21	S5 – C6	0.26
B2 – B3	-0.08	A2 – B3	-0.05	A2 – R3	0.15
B3 – B4	0.40	A3 – B4	0.10	A3 – R4	0.36
B4 – B5	0.03	A4 – B5	-0.04	A4 – R5	0.03
B5 – B6	-0.18	A5 – B6	0.00	A5 – R6	NA
R2 – R3	0.26	R2 – C3	-0.04	A2 – C3	0.48
R3 – R4	0.68	R3 – C4	0.08	A3 – C4	0.17
R4 – R5	-0.10	R4 – C5	0.09	A4 – C5	0.22
R5 – R6	NA	R5 – C6	-0.22	A5 – C6	0.59
C2 – C3	0.78			B2 – R3	0.21
C3 – C4	0.44			B3 – R4	-0.07
C4 – C5	0.70			B4 – R5	-0.16
C5 – C6	0.70			B5 – R6	NA
				B2 – C3	0.19
				B3 – C4	-0.19
				B4 – C5	0.13
				B5 – C6	-0.07

Formatting as in Table 9. 10

**Table 9:** Independent bivariate correlations between the accuracy on different trips in older adults

*r*_within−maze_	*r*_within−frame_	*r*_between−frames_
0.73	-0.11	-0.28
0.77	0.38	0.09
0.72	0.21	-0.07
0.67	0.16	-0.08
0.41	-0.09	0.01
0.48	0.11	-0.04
0.69	0.13	0.16
0.76	-0.05	0.28
-0.02	0.03	-0.03
-0.08	-0.02	-0.11
0.28	-0.08	0.01
0.26	0.00	-0.25
0.14	-0.11	0.03
0.23	0.31	-0.21
0.18	0.33	-0.10
0.16	0.05	-0.11
0.43		0.05
0.70		-0.13
0.55		-0.22
0.55		0.01
		0.31
		-0.03
		-0.01
		-0.17

**Table 10:** Independent bivariate correlations between the accuracy on different trips in young adults, supplementary data (3 s decision time)

*r*_within−maze_	*r*_within−frame_	*r*_between−frames_
0.59	0.08	0.38
0.63	0.20	-0.08
0.65	0.01	-0.10
0.69	0.26	0.12
0.78	-0.06	0.23
0.82	-0.16	0.27
0.85	0.01	0.09
0.75	0.21	0.27
0.13	0.09	0.37
0.07	0.30	0.42
0.29	0.14	0.34
-0.10	0.17	0.26
0.58	0.07	0.30
0.23	0.43	0.20
0.11	0.15	0.18
0.20	0.36	0.28
0.81		0.13
0.77		0.04
0.88		0.48
0.82		0.04
		0.06
		0.08
		0.11
		0.20

## Footnotes

1 Many correlations between self-guided trips did not fulfill the prerequisite of mutual independence called for by the subsequent statistical tests. Generally, if the correlation between A and B as well as that between B and C both are high positive, then the correlation between A and C must be at least moderately positive, hence it is not independent from the other two correlations.

## Declarations

### Open practices statements

The code for running the experiments and datasets generated and analyzed during the current study are available from the corresponding author on reasonable request.
